# Prucalopride inhibits the glioma cells proliferation and induces autophagy via AKT-mTOR pathway

**DOI:** 10.1186/s12883-018-1083-7

**Published:** 2018-06-04

**Authors:** Hong Qiao, Yong-Bo Wang, Yu-Mei Gao, Li-Li Bi

**Affiliations:** 1 Department of General Affairs Section, Second Affiliated Hospital of Mudanjiang Medical University, Mudanjiang, Heilongjiang 157009 People’s Republic of China; 2 Department of Respiratory Medicine, Second Affiliated Hospital of Mudanjiang Medical University, Mudanjiang, Heilongjiang 157009 People’s Republic of China; 3 Department of Medical Instruments, Second Affiliated Hospital of Mudanjiang Medical University, Mudanjiang, Heilongjiang 157009 People’s Republic of China

**Keywords:** Prucalopride, Glioma, Proliferation, Autophagy, AKT-mTOR pathway

## Abstract

**Backgrounds:**

Glioma is the most fatal primary brain glioma in central nervous system mainly attributed to its high invasion. Prucalopride, a Serotonin-4 (5-HT4) receptor agonist, has been reported to regulate neurodevelopment. This study aimed to investigate the influence of the Prucalopride on glioma cells and unveil underlying mechanism.

**Methods:**

In this study, glioma cells proliferation was evaluated by Cell counting kit-8 (CCK8). Wound healing and transwell assay were used to test cellular migration and invasion. Flow cytometry was utilized to determine cellular apoptosis rate. Apoptosis related markers, autophagy markers, and protein kinase B (AKT)-mammalian target of rapamycin (mTOR) pathway key molecules were detected using western blot assay.

**Results:**

As a result, the proliferation, migration and invasiveness of glioma cells were impaired by Prucalopride treatment, the apoptosis rate of glioma cells was enhanced by Prucalopride stimulation, accompanied by the increased pro-apoptosis proteins Bax and Cleaved caspase-3 and decreased anti-apoptosis protein Bcl-2. Prucalopride significantly promoted autophagy by increased expression level of Beclin 1 and LC3-II, while decreased expression level of p62. Prucalopride administration resulted in obvious inhibitions of key molecules of AKT-mTOR pathway, including phosphorylated- (p-) AKT, p-mTOR and phosphorylated-ribosomal p70S6 kinase (p-P70S6K).

**Conclusions:**

Taking together, these results indicate that Prucalopride may be likely to play an anti-tumor role in glioma cells, which suggests potential implications for glioma promising therapy alternation in the further clinics.

## Background

Glioma is known as the most frequently malignant primary brain tumor in humans, with annual incidence about 5 out of 100,000 persons [1, 2]. In spite of current progressive therapeutics for glioma, patients eventually yield this disease, seen from the poor overall survival of about 12–15 months [3]. Vigorous proliferation and uncontrollable invasion have been the barriers for clearing out glioma, and therapeutic options for which are groping, but most of these are limited. Accordingly, more efficient treatment strategies directing toward glioma have remained urgent presently.

Previous studies have suggested that the Serotonin (5-Hydroxytryptamine, 5-HT)-4 (5-HT_4_) receptor is richly distributed in the brain [4–6]. 5-HT_4_ receptors are identified as neurogenerative and neuroprotective actions, which are vital to maintenance of a normal enteric nervous system [6–8]. It is well known that activity of 5-HT_4_ receptors facilitates neurogenesis of injured enteric neuron and neural stem cells in the anastomotic ileum [9, 10]. Another study has documented that the 5-HT_4_ receptor represses the formation of neurites in neurons and relates with regulation of neural development [11, 12]. Activation of 5-HT_4_ receptors is demonstrated to improve memory performances [13]. Importantly, serotonin is reported to modulate some of cancer, namely placenta and choriocarcinoma cells [14], breast cancer cell [15], human prostate cancer tissue and cell [16]. Another report has demonstrated that a selective 5-HT_4_ receptor agonist, mosapride citrate inhibits the proliferative activity of human umbilical vein endothelial cells [17]. However, little is known about the effect of 5-HT4 receptor on glioma.

Prucalopride, a first-in-class dihydrobenzofuran-carboxamide derivative, is a highly selective, potent, and specific serotonin 5-HT_4_ receptor agonist with enterokinetic properties [18]. It is initially applied to treat constipation and other gastrointestinal disorders [19]. It is reported to exert a neuroprotection role in human neuroblastoma [20]. Prucalopride has been documented to associate with cholinergic neurotransmission [21]. Prucalopride is found to play a positively promotive function in stimulating Dopamine release which relieves depression or anxiety disorder [22]. Johnson et al. determined that Prucalopride increased acetylcholine level in prefrontal cortex [23]. In all, above observations indicates that Prucalopride regulates neurodevelopment. Nonetheless, it remains unclear whether and how Prucalopride is involved in the regulation of cell proliferation, metastasis and apoptosis in glioma cells.

Hence, the present study aimed to investigate the functional actions of Prucalopride on the proliferation, migration and apoptosis of human glioma cells as well as explore the possible mechanism underlying the anti-tumor effects of Prucalopride on glioma cells. Herein, the results of this study suggested that Prucalopride inhibited glioma cells proliferation and migration as well as induced apoptosis and autophagy, which was probably modulated by suppression of protein kinase B (AKT)- mammalian target of rapamycin (mTOR) signaling. These findings obtained from our investigations implicate that Prucalopride may be a potential therapeutic drug for the treatment of glioma.

## Methods

### Cell culture

Glioma U251 cell line was obtained from the Shanghai Institute for Life Science, Chinese Academy of Sciences (Shanghai, China). Glioma U87 cell line and normal human astrocyte SVG p12 were purchased from American Type Culture Collection (Manassas, VA, USA). Cells were cultured in RPMI-1640 medium supplemented with 10% fetal bovine serum (FBS), 100 U/ml penicillin and 100 μg/ml streptomycin (Invitrogen, Thermo Scientific, Waltham, MA, USA) at 37 °C under a humidified atmosphere of 5% CO_2_.

### Cell counting kit-8 (CCK-8) assay

Growth of the cell lines was evaluated using a CCK-8 (Beyotime Institute of Biotechnology, Shanghai, China) assay according to the manufacturer’s protocol. Cells (10^3^ cells/well) were seeded into 96-well plates and cultured for 24 h. Then, cells were incubated with different concentrations of Prucalopride (0, 0.1, 1, 10, 20, 50, 100 μM) and cultured for an additionally 72 h. For CCK-8 detection, 10 μl of CCK-8 solution was added into the wells and the cells were incubated at 37 °C for 1.5 h. The absorbance (optical density, OD) of cells was determined at 450 nm using a microplate reader (Bio-Rad, Hercules, CA, USA). The following experiment drug concentration (10 μM) was chosen from the series of the gradient concentration. Afterward, cells were incubated with Prucalopride (10 μM) and vehicle (DMSO, 0.1% in culture media) for 24, 48 and 72 h. Cells viabilities were measured by CCK-8 assay and OD values were measured as above described.

### Migration and invasion assays

After Prucalopride treatments for 48 h, cell capabilities of invasion and migration were examined by 24-well transwell chamber with or without Matrigel matrix (BD Bioscience, San Jose, CA, USA) following the instructions of the manufacturer. To evaluate cell migration, U251 cells (1 × 10^5^/ml) in 100 μl serum-free media were plated into top chamber of transwell migration chamber and 500 μl medium containing with 10% FBS was added into the lower chambers. Following incubation at 37 °C for 24 h, the filter was gently removed from the chamber and the remaining cells on the upper surface of the filter were wiped off with a cotton-tipped swab. The cells that had migrated on the lower surface of the filter were fixed with 4% paraformaldehyde for 10 min and stained with 0.5% crystal violet for 15 min. The number of migrated cells was counted from five random fields (× 200) under a microscope (Olympus, Tokyo, Japan). For invasion assay, transwell chamber was covered with Matrigel before a procedure similar to that for the migration assay was performed.

### Flow cytometry evaluation of apoptosis

To examine apoptosis, cells were double-stained with an Annexin V-Fluorescein isothiocyanate (FITC)/propidium iodide (PI) Apoptosis Detection kit (Beyotime Institute of Biotechnology) according to the manufacturer’s protocol. After Prucalopride treatments for 48 h, the cells were harvested, washed with cold phosphate-buffered saline (PBS), resuspended with binding buffer. Then cells were incubated with 5 μl Annexin-V-FITC reagent under room temperature for 5 min and were stained by PI reagent. The percentages of apoptosis cells were then analyzed by FACS Calibur flow cytofluorometry (BD Biosciences, San Jose, CA, USA) for the early apoptotic (Annexin V^+^ and PI^−^) and late apoptotic (Annexin V^+^ and PI^+^) cells. The apoptotic rate was determined using CellQuest software (BD Biosciences).

### Western blot analysis

After Prucalopride treatments for 48 h, cells proteins were extracted with radioimmunoprecipitation assay (RIPA) lysis buffer (Beyotime Institute of Biotechnology) and proteins concentrations were tested by a Bicinchoninic acid (BCA) protein assay (Beyotime Institute of Biotechnology). Following, equal amounts of protein were separated by 10% sodium dodecyl sulfate polyacrylamide gel electrophoresis (SDS-PAGE), and were transferred onto poly vinylidene difluoride (PVDF) membranes, then were blocked with 5% non-fat milk in Tris-buffered saline buffer with Tween-20. Afterwards, the PVDF membranes were incubated with primary antibody against LC3 (dilution 1:1, 000), Beclin-1 (dilution 1:1, 000), p62 (dilution 1:1, 000), AKT (dilution 1:1, 000), mTOR (dilution 1:1, 000), phosphorylated- (p-) AKT (dilution 1:1, 000), p-mTOR (dilution 1:1, 000) and phosphorylated-ribosomal p70S6 kinase (p-P70S6K) (dilution 1:1, 000) (Cell Signaling Technology, Inc., Danvers, MA, USA), GAPDH (Beyotime, dilution 1: 1000) and following with horseradish peroxidase-conjugated secondary antibodies (dilution 1:5, 000, Cell Signaling Technology, Inc.). Finally, proteins were visualized by enhanced chemiluminescence (ECL) kit (Millipore, Boston, MA, USA) and protein bands were measured with Image J software (National Institutes of Health (NIH), Stapleton, NY, USA).

### Statistical analysis

All data were expressed as means ± standard deviation (SD). Differences between the control and experimental results were tested by Student’s t-test (two-tailed) and one-way analysis of variance (ANOVA). A difference was considered statistically significant when the *P* value was deemed < 0.05 (*) or < 0.01 (**). All statistical analyses were carried out by SPSS version 22.0 (SPSS Inc., Chicago, IL, USA). All assays were performed three times.

## Results

### Prucalopride inhibited glioma cells growth

**Fig. 1:**
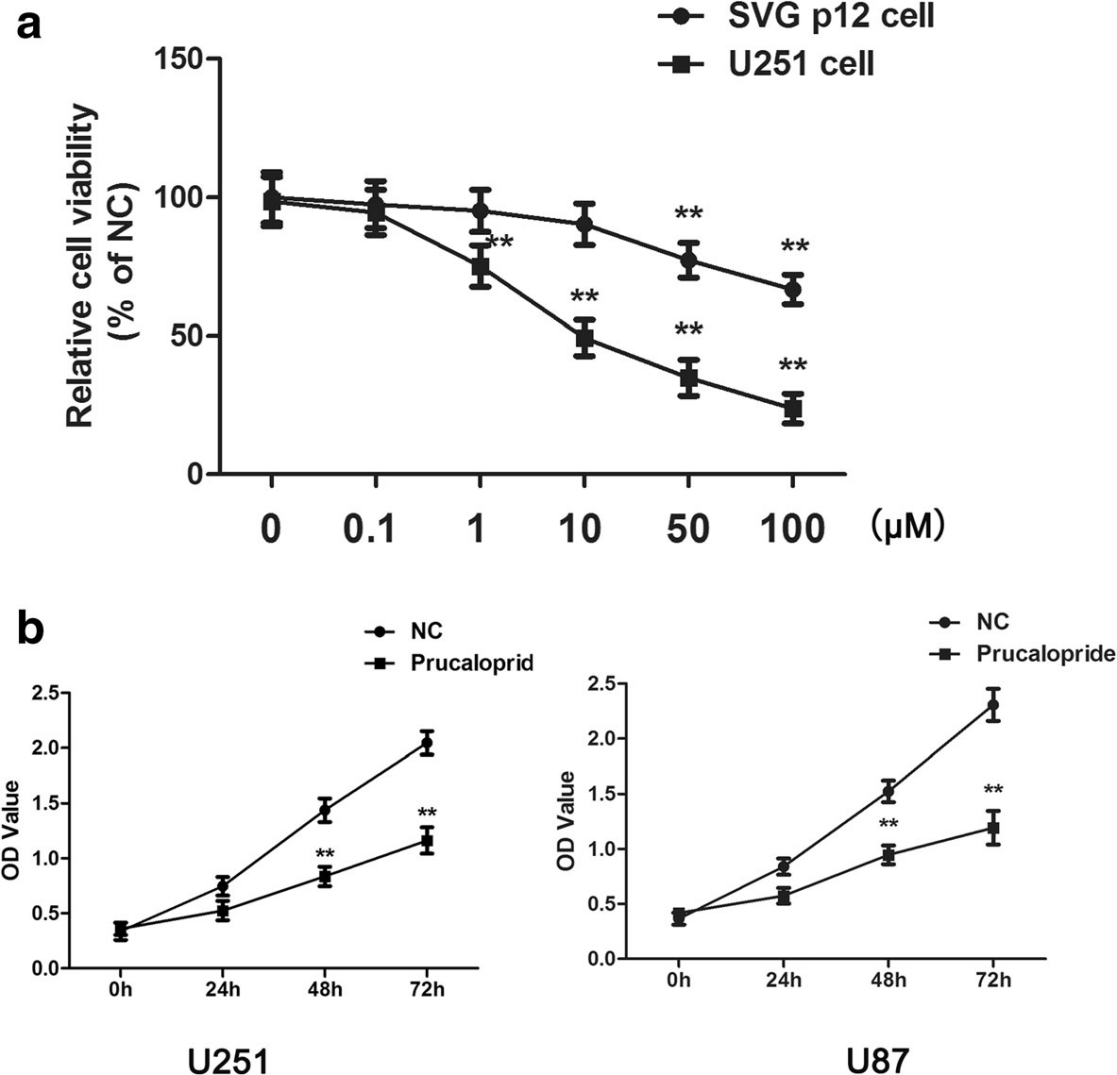
The suppressive effect of Prucalopride on glioma cells proliferation. **a** The viabilities of U251 and SVG p21 cells treated by Prucalopride with 0, 0.1, 1. 10, 50, 100 μM. ^**^P < 0.01 versus control group (0 μM Prucalopride group). **b** The OD values of U251 and U87 cells treated by Prucalopride with 10 μM (the following experiments were performed according to the concentration) after 0, 24, 48 and 72 h. The data are presented as the means ± SD, ^**^P < 0.01 versus NC group. Each assay was conducted in triplicate

To investigate the effect of Prucalopride on the proliferation of glioma cells, CCK-8 was conducted. A dose response in proliferation was observed as a decrease in U251 cells from 0.1 to 100 μM of Prucalopride, while the viability of SVG p12 cells was inhibited by Prucalopride beyond 50 μM (Fig. 1a). Consequently, 10 μM of Prucalopride was selected to perform following experiments. The IC_50_ value was 9.942 ± 0.346 μM. In addition, U251 and U87 cells proliferation was assessed by measuring the OD values at increasing time-points. As shown in Fig. 1b, OD values of U251 and U87 cells were significantly reduced as compared to untreated cells in a temporal manner. These results suggested that Prucalopride impeded growth of glioma cells.

### Prucalopride reduced glioma cells invasion and migration

**Fig. 2:**
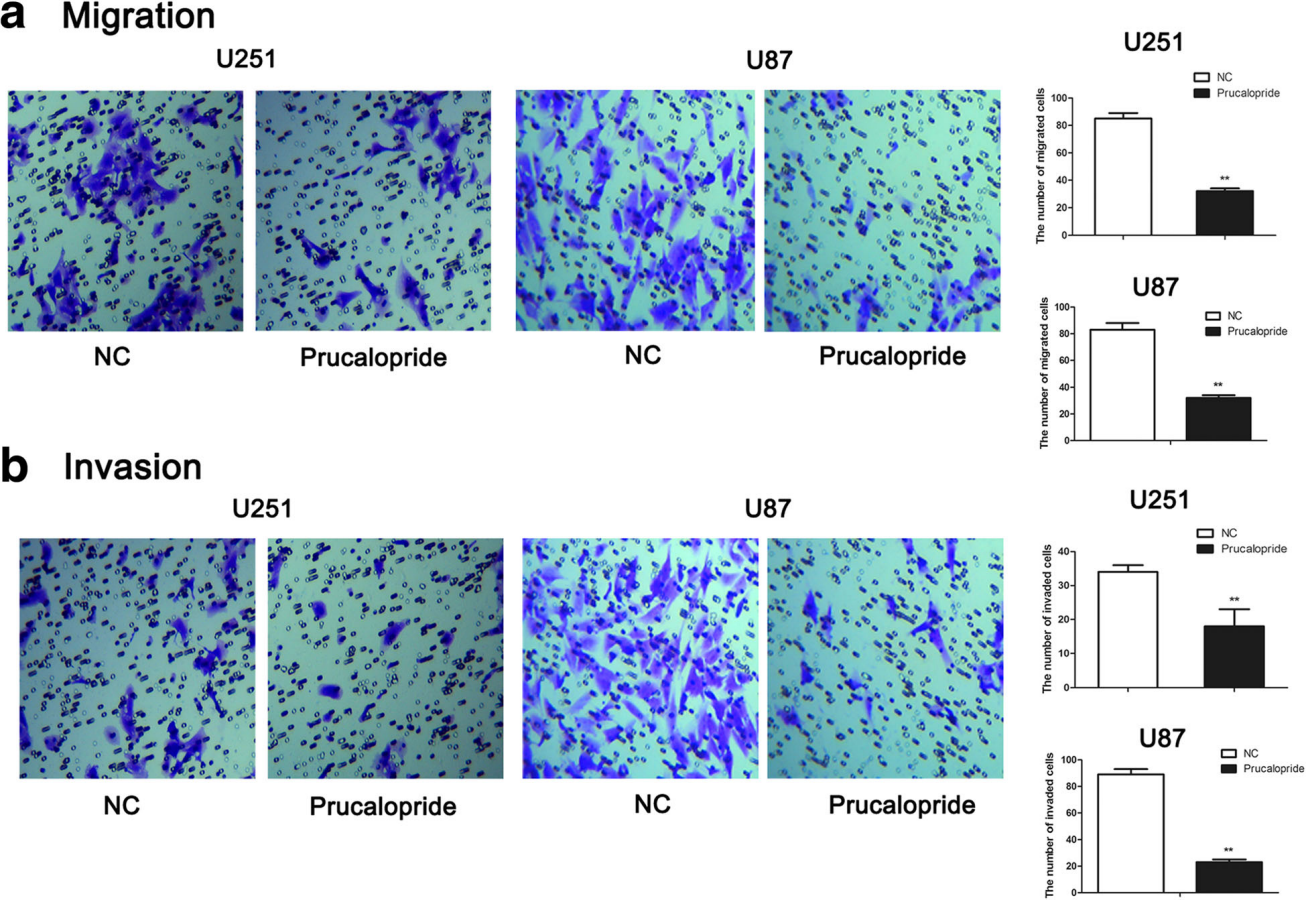
The capabilities of glioma cells migration and invasion were inhibited by Prucalopride. **a** The number of migrated cells was counted under a microscope (× 200) and quantitative analysis was shown in the right. **b** The number of invaded cells was counted under a microscope (× 200) and quantitative analysis was shown in the right. The data are presented as the means ± SD, ^**^P < 0.01 versus NC group. Each assay was conducted in triplicate

To examine the function of Prucalopride on glioma cells invasion and migration, transwell migration and invasion assays were performed. As shown in Fig. 2, the number of migrated cells was markedly decreased in Prucalopride group (32 ± 2) compared with NC group (85 ± 4) (*P* < 0.05). Similarly, the number of invaded cells was significantly suppressed in Prucalopride group (18 ± 5), as compared with NC group (34 ± 2) (*P* < 0.05). These data showed that the capabilities of glioma cells migration and invasion were inhibited by Prucalopride.

### Autophagy was induced by Prucaloprid

**Fig. 3:**
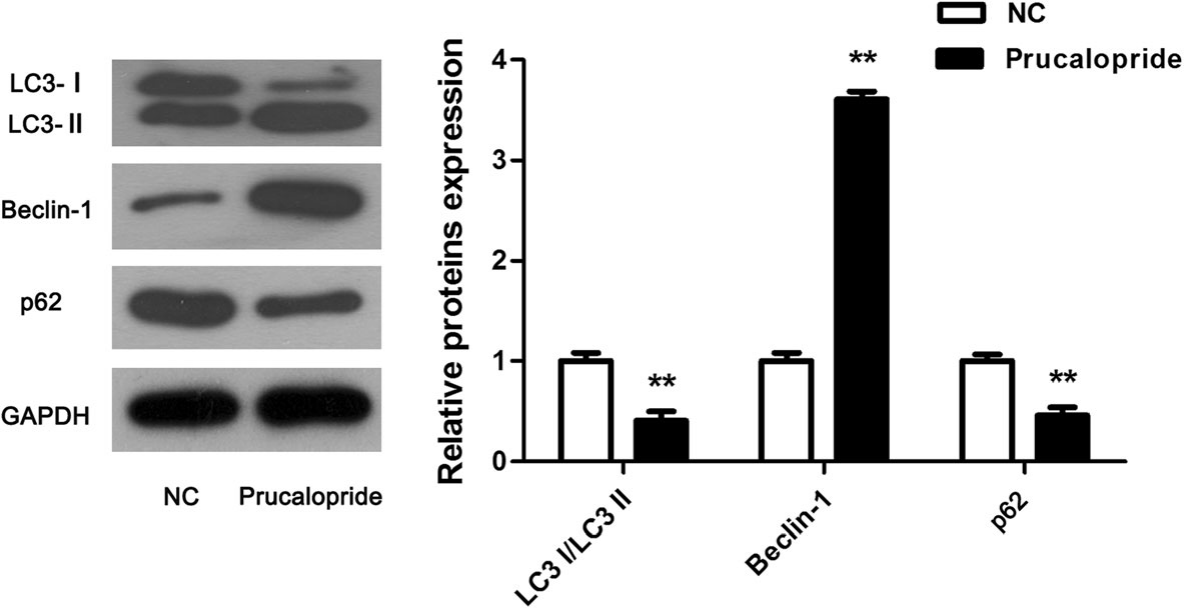
Induced effect of Prucalopride on glioma cells autophagy. Classic markers of autophagy were tested using western blot assay. The data are presented as the means ± SD, ^**^P < 0.01 versus NC group. Each assay was conducted in triplicate

To explore whether Prucaloprid affected autophagy in glioma cells, the classical autophagic markers, including Beclin-1, LC3- I/II and p62 were analyzed by western blot. Consistent with our predictions, Beclin-1 was observed to be upregulated while LC3-I/II and p62 was downregulated evidently (Fig. 3, *P* < 0.05), indicating that Prucaloprid promoted autophagy in glioma cells.

### Prucaloprid induced glioma cells apoptosis

**Fig. 4:**
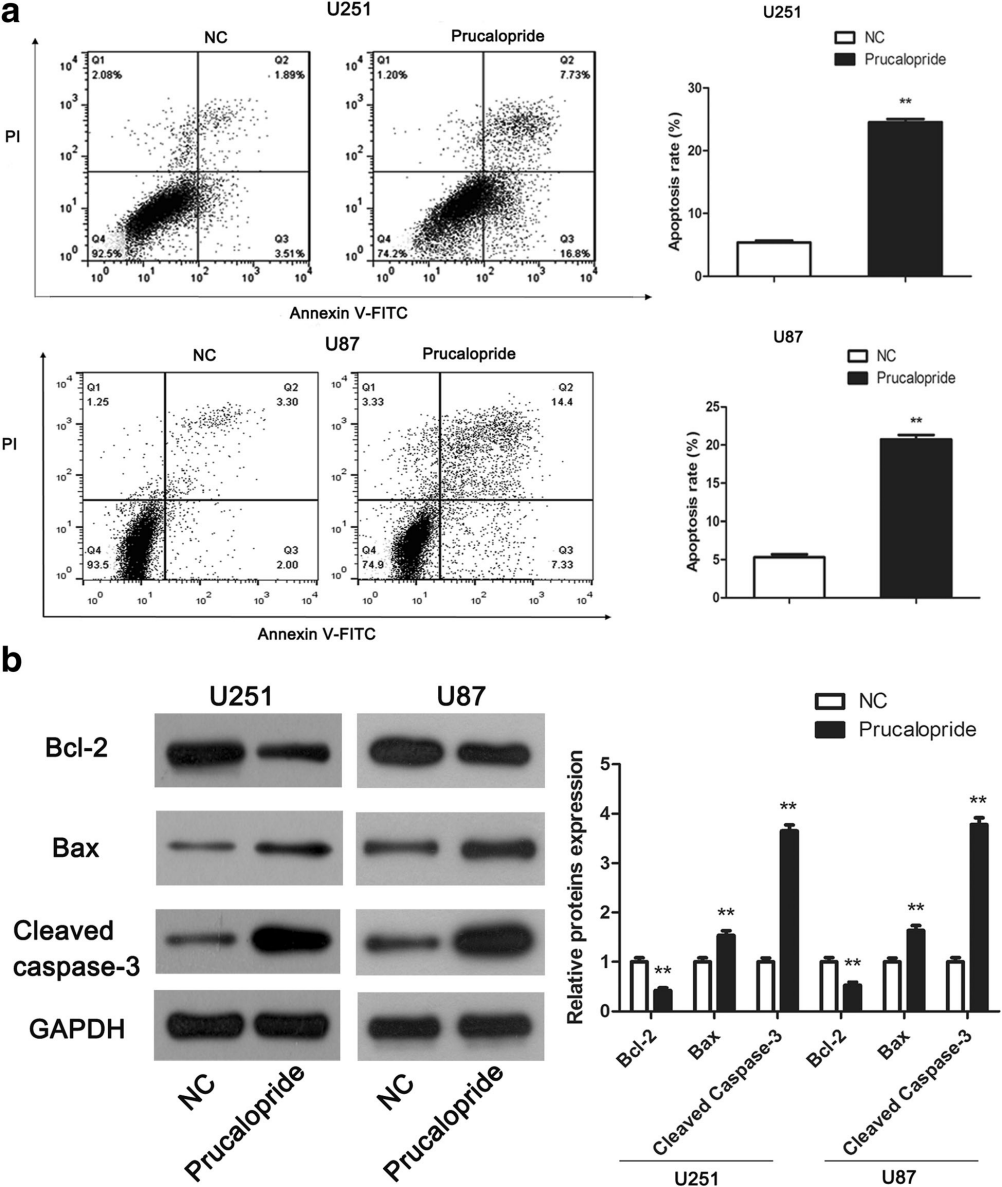
The stimulative effect of Prucalopride on glioma cells apoptosis. **a** Changes in glioma cells apoptosis rates were measured by flow cytometry. **b** Apoptosis related markers were detected by western blot assay. The data are presented as the means ± SD, ^**^P < 0.01 versus NC group. Each assay was conducted in triplicate

To define the role of Prucalopride on glioma cells apoptosis, flow cytometry assay and western blot assay were carried out. As showed in Fig. 4a, the apoptosis rates of U251 and U87 cells in Prucalopride group was increased significantly in comparison with NC group (*P* < 0.05). Molecularly, we further examined the apoptosis related markers, namely Bcl-2, Bax and Cleaved caspase-3 using the western blot assay. The levels of anti-apoptosis protein Bcl-2 was obviously downregulated while pro-apoptosis proteins Bax and Cleaved caspase-3 were markedly upregulated in Prucalopride group compared with NC group (Fig. 4b, *P* <  0.05). Above of these results suggested that Prucalopride induced apoptosis of glioma cells.

### AKT/mTOR activity was influenced by Prucaloprid

**Fig. 5:**
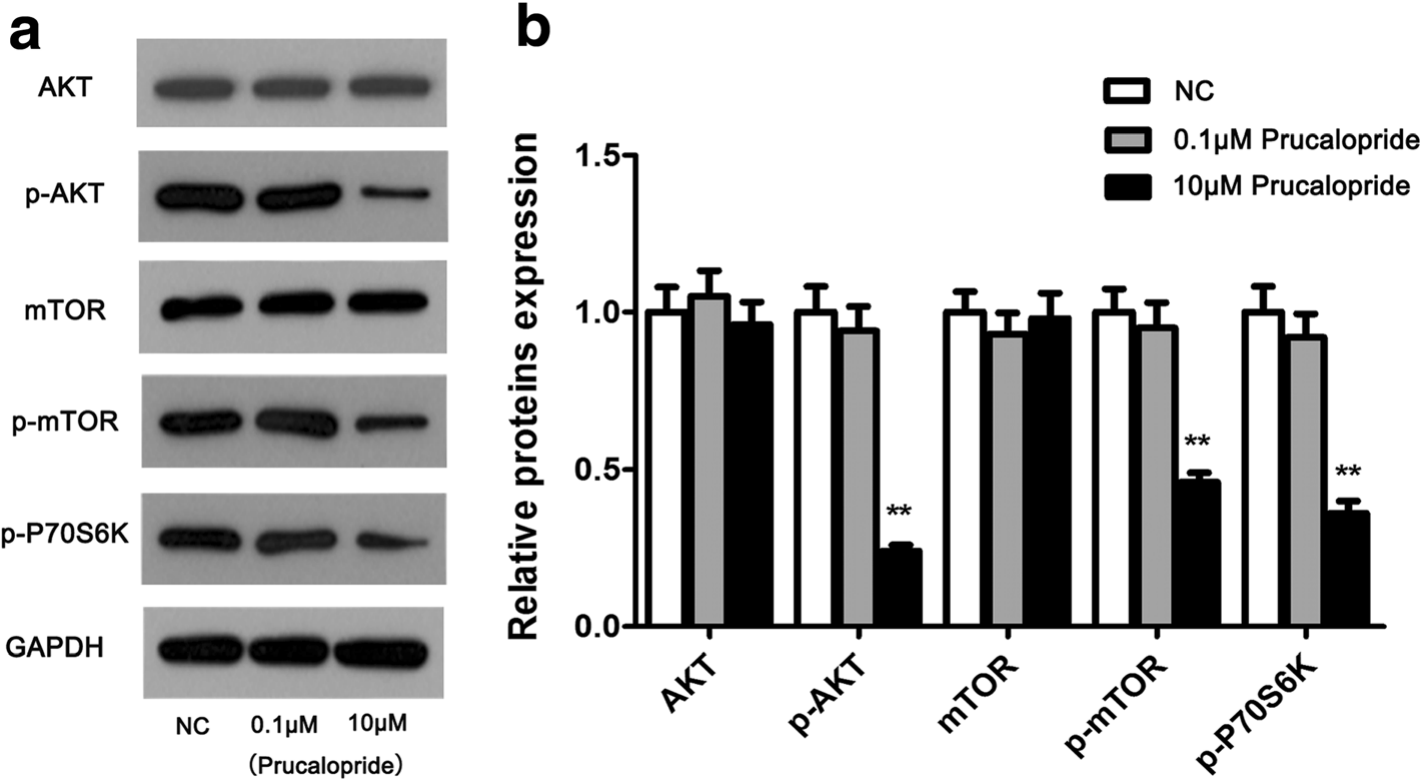
Activity of AKT/mTOR signaling was suppressed by Prucalopride. **a** AKT/mTOR signaling key proteins were examined using western blot assay. **b** Densitometry analysis of proteins bands. The data are presented as the means ± SD, ^**^P < 0.01 versus NC group. Each assay was conducted in triplicate

To gain insight into the molecular mechanism by which Prucalopride regulated glioma cell proliferation and migration, we thus tested the effect of Prucalopride on the activation of AKT/mTOR signaling pathway in U251 cells using western blot assay. To rule out the effect of Prucalopride dose on AKT/mTOR signaling pathway, 0.1 μM concentration was selected as the comparison of dose impact. As shown in Fig. 5, the levels of p-AKT and p-mTOR were decreased while total of AKT and mTOR were no detectable changes in 10 μM Prucalopride group on comparing the NC and 0.1 μM Prucalopride groups (*P* < 0.05, *P* > 0.05 respectively). Moreover, the p-P70S6K level was downregulated in Prucalopride group compared with NC and 0.1 μM Prucalopride groups (*P* < 0.05). Whereas levels of proteins in Prucalopride groups were no significant differences compared with those in NC group. These data suggested that AKT/mTOR signaling may involve in the anti-tumor effect of Prucalopride in glioma cells.

## Discussion

In the current study, we demonstrated that Prucalopride played an inhibiting role in glioma cells proliferation, migration and invasion as well as a promoting effect in glioma cells apoptosis. In addition, autophagy was induced by Prucalopride treatment. Furthermore, the activity of AKT-mTOR signaling was suppressed by Prucalopride. Collectively, these data suggest Prucalopride might be a potential clinical application for glioma.

The regulation of tumor cell proliferation, migration, invasion and apoptosis by Prucalopride, a 5-HT_4_ receptor agonist, has been investigated in this study. The OD values of glioma cells were decreased by administration of Prucalopride. The number of migrated and invaded glioma cells was reduced by Prucalopride treatment. Further, it was observed that apoptosis rate of glioma cells was increased after Prucalopride treatment, in support of this, the pro-apoptosis markers Cleaved caspase-3 and Bax were up-regulated while anti-apoptosis marker Bcl-2 was down-regulated by Prucalopride. A previous research by Takeshi et al. demonstrated that another Selective 5-HT4 Receptor Agonist, mosapride citrate also exerted antiangiogenic and anti-proliferative effects of human umbilical vein endothelial cells [17]. Another study showed that 5-HT_4_ receptor acted as a tumor suppressor role in ovarian carcinogenesis [24]. All in all, these evidence indicate that Prucalopride performs a potential anti-tumor role in glioma.

Autophagy is a highly conserved catabolic process that captures, degrades, and recycles damaged organelles, waste macro-molecules and other substances in cells [25, 26]. It is considered as a survival mechanism conventionally improves the survival of cells [27]. Autophagy can be activated in many types of cells, including gliomas [28]. However, paradoxically, it is increasingly being applied to facilitate cancer cell death, called autophagic or type II programmed cell death [29, 30]. To explore whether autophagy mediates anti-tumor action of Prucalopride in glioma, in this study, markers of autophagy were examined. The results showed that and Beclin-1 was increased while LC3-I/II and p62 were decreased by Prucalopride administration. Our investigations, in combination with previous studies, suggest that autophagy is induced by Prucalopride in glioma, implying a significant role of autophagy involved in tumor-inhibitory role by Prucalopride.

To gain some insight into the molecular mechanisms of Prucalopride-mediated anti-cancer action in glioma cells, the AKT-mTOR signaling pathway was assessed. It is well-established that the AKT-mTOR signaling pathway plays a crucial role in the regulation of cell proliferation and cell survival in wide array of tumor cells, usually linked with oncogenesis, including gliomas [31–33]. PI3K phosphorylation and its product phosphorylated- phosphatidylinositol 3,4,5-triphosphate (p-I3-P) with AKT binding site, thus AKT is phosphorylated. The p-AKT activated mTOR phosphorylation, which is followed by the down-regulation of phosphorylated p70S6K, a major effector of mTOR in the downstream of the mTOR signaling pathway [34]. Down-regulation of phosphorylated p70S6K induces autophagy [35]. Thereby, the PI3K/AKT/mTOR/p-P70S6K has been known to be frequently implicated to modulate the initiation of autophagy [36–38]. It was reported that autophagy mediated of suppressing glioma cells proliferation via involvement of this signaling [39]. Yu-Chen et al. revealed that autophagy formation was participated in the glioma cells growth inhibition through regulating this signaling [40]. Overall, above data demonstrated that the AKT-mTOR signaling was involved in the anti-tumor action of Prucalopride on glioma cells. In spite of this, limitations of the current work should be acknowledged that this study has examined merely U251 and U87 cells, further studies are needed to illuminate the molecular basis involved in this process through more cell lines and even animal model construction.

## Conclusions

In conclusion, the findings of the present study suggested that Prucalopride exerted anti-proliferative, anti-migratory and anti-invasive effects in glioma cells, aside from this, it also developed an inductive action of autophagy occurrence on glioma cells. These effects were impelled by Prucalopride possibly via inhibiting AKT-mTOR signaling. This work indicates Prucalopride might function as a cancer suppressive agent for glioma, implying Prucalopride would be a promising candidate drug treatment for glioma in the further clinic.

## Abbreviations

5-HT4: Serotonin-4
AKT: Protein kinase B
Annexin V-FITC/PI: Annexin V-Fluorescein isothiocyanate/propidium iodide
ANOVA: One-way analysis of variance
mTOR: Mammalian target of rapamycin
OD: Optical density
P70S6K: Ribosomal p70S6 kinase
SD: Standard deviation
SDS-PAGE: Sulfate polyacrylamide gel electrophoresis
